# Paquinimod‐hydrogel hybrid microneedle array patch alleviates hypertrophic scar via inhibiting M1 polarization

**DOI:** 10.1002/btm2.70016

**Published:** 2025-03-15

**Authors:** Zihui Zhang, Peng Wang, Hengdeng Liu, Hanwen Wang, Miao Zhen, Xuefeng He, Suyue Gao, Juntao Xie, Julin Xie

**Affiliations:** ^1^ Department of Burns and Wound Repair The First Affiliated Hospital of Sun Yat‐sen University Guangzhou Guangdong Province China; ^2^ Department of Pediatric Surgery The First Affiliated Hospital of Sun Yat‐sen University Guangzhou Guangdong Province China

**Keywords:** hypertrophic scar, macrophage polarization, microenvironment, paquinimod‐hydrogel hybrid microneedle array patch

## Abstract

Hypertrophic scar (HS) is one of the most common complications of skin injuries, with a lack of effective therapeutic approaches to date. Most current research has focused on the dysfunction of hypertrophic scar fibroblasts (HSFBs) and dermal vascular endothelial cells (HDVECs), neglecting the crucial role of the inflammatory microenvironment that causes them to be abnormal. In this study, we first discovered and validated that the S100A8/9 specific inhibitor Paquinimod could inhibit macrophage polarization toward M1, and further suppress the proliferation, migration, collagen formation, and angiogenesis of HSFBs and HDVECs in vitro. This mechanism has also been validated in a rat model of HS. Then, we developed a good biocompatibility and penetrability Paquinimod‐Hydrogel Hybrid Microneedle Array Patch (PHMAP) for HS treatment. With the advantages of excellent penetrability, surface sealing, sustained release, and precise uniform distribution, PHMAP exhibited superior therapeutic efficacy over intravenous and intradermal injections. These results suggest that PHMAP can be a promising and advanced solution for HS prevention and therapies.

**Translational impact statement**

We developed the Paquinimod‐Hydrogel Hybrid Microneedle Array Patch (PHMAP), a safe, user‐friendly transdermal solution for hypertrophic scarring, enabling early self‐administered treatment to enhance adherence, reduce medical burden, and provide both preventive and therapeutic benefits.

## 1 INTRODUCTION

Hypertrophic scar (HS) is abnormal wound healing following trauma or surgical intervention and usually leads to aesthetic and functional impairments for patients.^1^, ^2^, ^3^, ^4^, ^5^ Although currently therapeutic approaches are various, including compression therapy, silicone‐based agents, glucocorticoids, surgery, and radiation therapy, their overall efficacy remains limited. The main reasons are the lack of precise treatments targeting the pathogenesis and the inefficient drug delivery into the scar tissue.^6^, ^7^, ^8^, ^9^, ^10^ Consequently, developing new, efficient, and precise therapeutic approaches is crucial.

Despite the complexity of HS pathogenesis, the dysfunction and hyperactivation of human hypertrophic scar fibroblasts (HSFBs) and dermal vascular endothelial cells (HDVECs) are recognized as critical contributors to HS development.^11^, ^12^, ^13^ However, most current research focuses primarily on the biological characteristics and functions of HSFBs and HDVECs, while neglecting the role of the HS microenvironment that drives these cellular abnormalities.^14^, ^15^ Recent studies and clinical observations have confirmed that a sustained inflammatory response is the dominant factor leading to the development of HS. Therefore, it is urgent to develop a precise, efficient HS therapeutic method targeting its inflammatory microenvironment.^16^

Emerging research indicates that, unlike in atrophic scars where IL‐8 overactivity causes neutrophils to release lysosomal enzymes,^17^ the cascade response triggered by excessive macrophage polarization toward the M1 phenotype during wound healing and early scar formation may play a key role in the sustained inflammatory response in HS.^18^, ^19^ As significant members of the calcium‐binding protein family, S100A8 and S100A9 modulate macrophage polarization and proliferation via the TLR4/MyD88/NF‐κB signaling axis.^20^, ^21^, ^22^, ^23^, ^24^ Therefore, effective inhibition of S100A8/A9 in HS could potentially enhance the therapeutic outcomes, providing a promising strategy for improving HS treatment.

Paquinimod (ABR 25757), a specific inhibitor of S100A9 that blocks its interaction with the TLR4 receptor, gained significant attention during the COVID‐19 pandemic in 2020.^25^ It has also been investigated for its therapeutic potential in conditions such as inflammatory bowel disease^26^ and diabetes mellitus.^27^ In systemic sclerosis, Paquinimod reduces the expression of CCL2, TGF‐β, and other markers, exhibiting clear anti‐fibrotic and anti‐inflammatory effects.^28^, ^29^ Despite these findings, its application in treating skin disorders, including HS, remains unexplored. Clinical trials have predominantly used oral administration, and animal studies typically involve oral delivery (via drinking water or gavage) or intraperitoneal injection.^26^, ^30^, ^31^ This study aims to advance the field by comparing various local administration methods—intravenous injection, subcutaneous injection, and microneedle delivery—to optimize Paquinimod's therapeutic efficacy.

The prolonged HS treatment cycle makes systemic immunomodulatory drugs prone to high side effects, while localized injections are time‐consuming, costly, and have low patient compliance.^32^, ^33^, ^34^, ^35^ Thus, developing a drug delivery method with mild, stable, and long‐lasting effects is essential. An ideal wound dressing in early healing should provide hemostasis, adhesion, and exudate absorption, while also ensuring biocompatibility, mechanical integrity, and easy removal.^36^ Hydrogels, known for sustained drug release, seal wounds, promote healing, aid debridement, and reduce inflammation.^37^, ^38^, ^39^ By integrating hydrogels with drugs, microneedles enable precise delivery through the stratum corneum.^40^, ^41^ The patch adheres to the HS surface, reduces water loss, penetrates the dermis for precise delivery, and dissolves gradually for slow, sustained release, enhancing treatment efficacy and reducing administration frequency.

**SCHEME 1:**
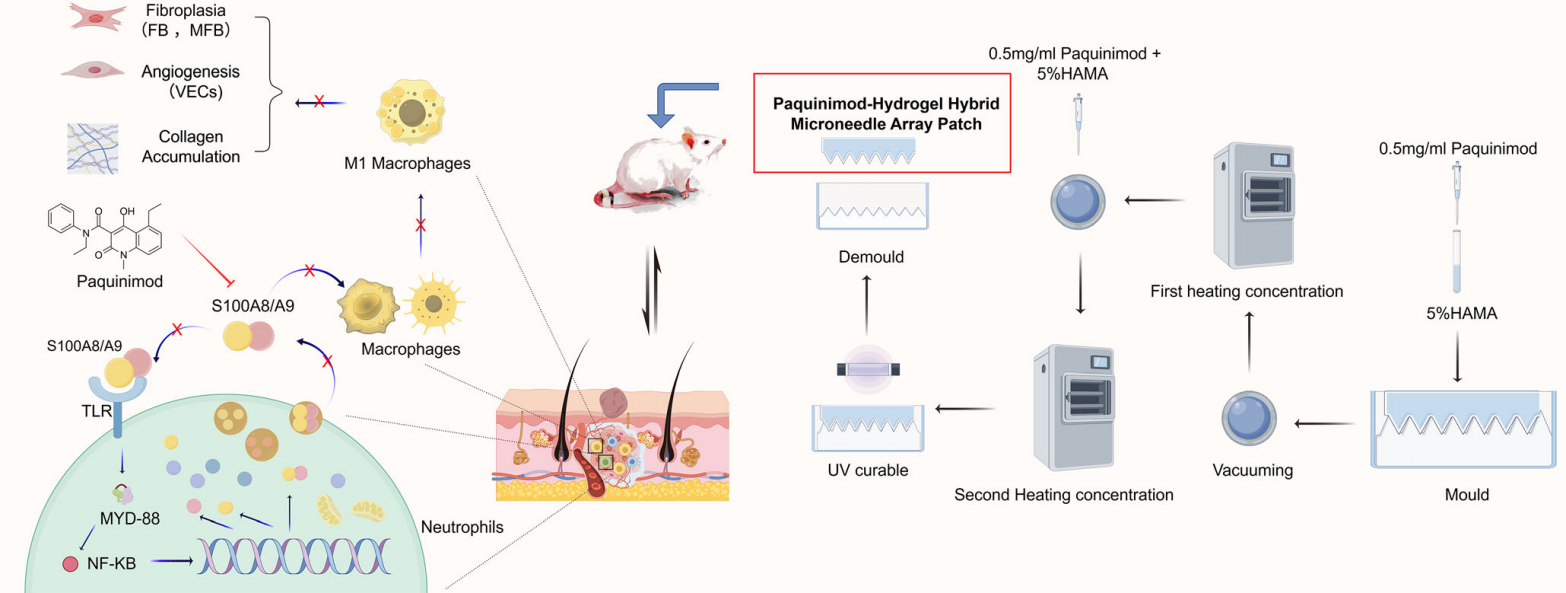
Schematic representation of the preparation and application of PHMAP for treating HS in rat tails. The microneedles were fabricated using a Paquinimod‐HAMA hydrogel precursor solution, which was concentrated through repeated defoaming and light curing. PHMAP specifically targets S100A8/A9, inhibiting macrophage polarization toward the M1 phenotype. This inhibition suppresses the proliferation, migration, collagen synthesis, and angiogenesis of HSFBs and HDVECs, leading to significant HS alleviation.

In this study, we developed a Paquinimod‐Hydrogel Hybrid Microneedle Array Patch (PHMAP) using methacryloylated hyaluronic acid (HAMA) hydrogels combined with Paquinimod and comprehensive validation of its mechanism and efficacy in the treatment of HS. We first discovered and validated in vitro that Paquinimod precisely attenuates the inflammatory response primarily by inhibiting macrophage polarization toward M1, which in turn inhibits HSFBs and HDVECs. This mechanism has also been validated in a rat model of HS. With the advantages of excellent penetrability, surface sealing, sustained release, and precise uniform distribution, PHMAP exhibited superior therapeutic efficacy over intravenous and intradermal injections. These findings suggest that PHMAP could be a promising and technically advantageous HS prevention and therapy agent (Scheme 1).

## 2 METHODS

### 2.1 Microneedle mechanics test

Secure the microneedle onto the moving sensor surface of the Microneedle Strength Testing Machine (Shanghai Baosheng TA.XTC‐20, China) in such a way that the axis of the microneedle is perpendicular to the direction of the applied force. Initiate the machine to move the sensor surface at a constant speed, thereby applying an axial force to the microneedle that is perpendicular to its axis. Throughout the stress application process, the force‐displacement curve of the microneedle is continuously recorded.

### 2.2 Drug release profile of PHMAP in PBS

To investigate the drug release profile of PHMAP, a sample was immersed in 50 mL of PBS at room temperature. The system was subjected to continuous shaking to ensure uniform distribution of the drug. At regular intervals of 30 min, 1 mL of the medium was withdrawn and immediately replaced with an equivalent volume of fresh PBS. The concentration of Paquinimod in the medium was quantitatively analyzed using a high‐performance liquid chromatography (HPLC) system (Agilent 1260 Infinity II, USA).

### 2.3 Live/dead cell viability assay

RAW264.7, HSFB, and HDVEC cells were seeded into 6‐well plates (5 × 10^4^ cells per well) and co‐cultured with fresh medium containing various test samples, including PBS, Paquinimod (500 μg/mL), S100A8 (50 μg/mL), and HAMA (5% w/v), 48 h later, the same mixture was changed. After 4 days of culture, live and dead cells were assessed using the Calcein‐AM/PI Live/Dead Cell Double Staining Kit (Abbkine). The cells were observed using a fully automated inverted fluorescence microscope (Olympus IX83, Olympus, Japan). Cell viability was calculated using the formula: Cell viability (%) = (*C*_Calcein‐AM+_/*C*_all_) × 100, where *C*_Calcein‐AM+_ represents the number of Calcein‐AM‐positive cells and *C*_all_ represents the total number of cells.

### 2.4 In vitro cell viability assay

HSFB and HDVEC cells were seeded into 96‐well plates (3 × 10^3^ cells per well) and co‐cultured with fresh medium containing various test samples: PBS, Paquinimod (20, 50, 100, and 500 μg/mL). After 48 h of culture, cell viability was assessed using the Cell Counting Kit‐8 (BS350A, CCK‐8, Biosharp) assay. Optical density (OD) was measured at 450 nm using a full‐wavelength zymography microplate reader (Thermo, USA). Data represent the results of five independent experiments.

### 2.5 In vitro cell migration assay

HSFB and HDVEC cells were seeded into 6‐well plates (2 × 10^6^ cells per well). A 200 μL sterile pipette tip was used to create a scratch in the center of the confluent cell monolayer. The wells were then washed with PBS and treated with fresh serum‐free medium containing various test samples: PBS, Paquinimod (50 and 100 μg/mL). Subsequently, phase‐contrast images of the wound closure were captured at 0, 6, 12, 24, and 48 h using an inverted microscope (OLYMPUS, CKX, Japan). The migration rate was calculated by Image J software with the following formula: Migration rate (%) = (*d*_0_ − *d*_*n*_)/*d*_0_ × 100%, where *d*_0_ represents the original Scratch width (*t* = 0 h) and *d*_*n*_ represents the residual Scratch width at the indicated time point (*t* = *n h*).

### 2.6 Macrophage products collection and indirect co‐culture with HSFBs and HDVECs

Macrophages(RAW264.7) treated with fresh serum‐free medium and PBS, LPS (100 ng/mL), and LPS (100 ng/mL) plus Paquinimod (100 μg/mL) for 24 h. The culture supernatants were collected, centrifuged to remove cell debris, and stored. HSFBs and HDVECs were harvested, counted, and seeded. After 4 h to allow for cell adhesion, the macrophage products were added in. The co‐culture was maintained at 37°C and 5% CO2. The methods of Cell Viability Assay and Cell Migration Assay are the same as 2.4 and 2.5.

### 2.7 In vitro collagen contraction experiment

Collagen solution (CELL BIOLABS, CBA‐5020, USA) was added to 24‐well plates and allowed to cure at 37°C for 1 h. HSFBs were then inoculated into the collagen matrix and cultured with fresh serum‐free medium containing various test samples: PBS, LPS (100 ng/mL), and LPS (100 ng/mL) + Paquinimod (100 μg/mL), inducing macrophage cell products at 37°C for 48 h. The contraction of the collagen matrix was subsequently observed. Images of the collagen matrix were captured, and Image J software was utilized to quantify and analyze the contraction effects of the cells on the matrix.

### 2.8 In vitro angiogenesis assay

HDVECs were inoculated into 24‐well plates coated with Matrigel (Beyotime, C‐0372, China) at an appropriate density and cultured in a CO₂ incubator with fresh medium containing various test samples, including PBS, LPS (100 ng/mL), and LPS (100 ng/mL) + Paquinimod (100 μg/mL) induced macrophage cell products. Cell morphology was periodically observed under light microscopy. After 4 and 8 h, images of the cells were captured using an Olympus CKX microscope (OLYMPUS, Japan). The numbers of branches, junctions, meshes, and segments of vessels were quantified in five random fields of view using ImageJ software, Angiogenesis Analyzer.

### 2.9 Macrophage polarization induction assay

RAW264.7 cells were seeded into 6‐well plates (2 × 10^6^ cells per well). Fresh medium containing various test samples (PBS, LPS (100 ng/mL), LPS (100 ng/mL) + Paquinimod (100 μg/mL), and Paquinimod (100 μg/mL)) was added, and the cells were co‐cultured for 48 h. The medium was then aspirated, the cells were digested, and collected by resuspending them in 1 mL of PBS.

### 2.10 Detection of M1‐type and M2‐type macrophages by flow cytometry tests

The cell suspension was centrifuged at 5000 rpm for 5 min. The cells were then incubated with 0.5 μL of F4/80 (PE Rat Anti‐Mouse F4/80 [T45‐2342]), CD86 (PE‐Cy7 Rat Anti‐Mouse CD86 [GL1]), and CD206 (Alexa Fluor 647 Rat Anti‐Mouse CD206 [MR5D3]) (BD Science, UK) for 15 min at room temperature. Following incubation, the cells were fixed in PBS and analyzed using an ultra‐high‐speed flow cytometer (Invitrogen Attune NXT, Thermo, USA). The results were analyzed using FlowJo software.

### 2.11 Detection of M1‐type and M2‐type macrophages by qPCR test

Macrophage total RNA was extracted and reverse transcribed to obtain cDNA, and primers were designed according to the gene sequences of mouse CD86, F4/80, CD163, CD11b, IL‐10, iNOS, CD68, and CD206 recorded in Genbank, and the surface markers and cytokines of macrophages were analyzed quantitatively at the transcript level by using a real‐time quantitative PCR instrument.

### 2.12 Establishment of HS model

Eight‐week‐old adult male Sprague–Dawley (SD) rats were obtained from Wuhan Beinlai Bio (Wuhan, China). All procedures involving animals were approved by the Animal Care and Use Committee of the First Affiliated Hospital of Sun Yat‐sen University (approved animal protocol number: [2022]025). We confirm that our animal study strictly adheres to the ARRIVE guidelines. An 8 × 8 mm full‐thickness square excision wound was created on the tail of each rat, which was then covered with dry sterile gauze. A 2 cm‐diameter stainless steel ring was fixed around the tail to maintain constant tension.^42^ The HS model was considered successfully established approximately 21 days after the initial wound formation.

### 2.13 Treatment regimen and efficacy assessment of HS

Each rat was assigned a unique number (1–35), and a computer‐generated random number sequence was used to allocate them into five experimental groups of seven rats each. The treatment included injections of 1 mL of 0.5 mg/mL Paquinimod PBS solution, 1 mL of PBS solution, 0.1 mL of 5 mg/mL aqueous Paquinimod PBS solution, and 0.1 mL of 50 μg/mL S100A8 PBS solution. Additionally, a PHMAP containing 500 μg of Paquinimod was applied to treat the caudal HS. The direction of injection was parallel to the long axis of the scar. Scar area, scar volume, thickness, and scoring were assessed weekly. On day 21, all rats were necropsied, and their livers and scar tissues were removed for further analysis.

### 2.14 In vivo histopathological and immunofluorescence analyses

On day 21, scar samples were fixed overnight in 4% paraformaldehyde (PFA), embedded in paraffin, and sectioned into 5 μm thick slices. The scar elevation index (SEI) and collagen volume fraction (CVF) were evaluated using Hematoxylin and Eosin (HE) and Masson's trichrome staining. The SEI was calculated as the height of the tissue in the scar area divided by the height of the adjacent normal tissue. The CVF was calculated as the percentage of the collagen‐positive area relative to the total tissue area, expressed as a percentage (CVF (%) = (collagen‐positive area / total tissue area) × 100).

### 2.15 Double immunofluorescence staining was performed to detect cells, including iNOS/CD68, Collagen I/Ki‐67, and CD31/Ki‐67

DAPI was used to counterstain nuclei. Briefly, paraffin‐embedded sections (5 μm) were deparaffinized and blocked with 5% serum. The sections were then incubated at 4°C with the following primary antibodies: rabbit anti‐CD68 (1:500, GB113109, Servicebio), rabbit anti‐iNOS (1:500, GB11119, Servicebio), rabbit anti‐COL1A1 (1:250, ab138492, Abcam), rabbit anti‐CD31 (1:500, ab182981, Abcam), and mouse anti‐Ki‐67 (1:500, ab279657, Abcam). After three washes with PBS, the sections were incubated with fluorescent secondary antibodies (1:2000, ab150120, ab150077, Abcam) for 1 h, followed by DAPI staining for 10 min at room temperature. The stained sections were then examined using an inverted fluorescence microscope (IX83, Olympus, Japan).

### 2.16 Type I and type III collagen area measurement by sirius red staining

The tissue was fixed and sectioned, then subjected to Sirius red staining. Post‐staining, the sections were examined under a polarized light microscope and imaged. Under polarized light, Type I collagen appeared orange‐yellow or red, while Type III collagen appeared green. The images were then processed using image analysis software (e.g., ImageJ). By adjusting the threshold, the software differentiated the two types of collagen, measured their respective areas, and calculated the area ratio of Type I to Type III collagen, thereby quantitatively assessing the collagen distribution within the tissue.

### 2.17 In vivo evaluation of drug side effects

Liver samples were collected from day 21 rats, fixed in 4% PFA overnight, embedded in paraffin, and cut into 5 μm thick sections. Pathologic changes and the extent of damage were assessed by HE staining.

### 2.18 Statistical analysis

All results are expressed as mean ± SD unless otherwise stated. Comparison of expression differences between control and experimental groups was performed by two‐tailed unpaired Student's t‐test. Comparisons of differences between multiple groups were performed using one‐way analysis of variance (ANOVA). All statistical analyses were performed using GraphPad Prism 10.1.2 software. *p* < 0.05 indicates significant differences. (Unless otherwise specified, *p < 0.05, **p < 0.01, compared with the control group; ^#^ *p* < 0.05, ^##^ *p* < 0.01, compared with the PHMAP group; NS, no significant difference).

## 3 RESULTS AND DISCUSSION

### 3.1 Synthesis and Characteristic of PHMAP

Long‐term continuous systemic application poses risks such as immune abnormalities, increased treatment costs, reduced patient compliance, and potentially decreased treatment efficacy.^43^ The natural barrier of the skin's stratum corneum limits the efficiency of most transdermal drug delivery systems.^44^ Therefore, traditional topical application often struggles more to penetrate thickened or fibrotic dermal layers.^45^ Microneedles, however, offer a more reliable and efficient means of delivering drugs to targeted cells within the skin, thereby enhancing therapeutic outcomes.^46^ To address this challenge and explore a gentler, more precise, and sustained treatment method, we developed the HAMA‐based Hybrid Microneedle Array Patch. This is particularly crucial for drugs like paquinimod, which require controlled delivery into the deeper layers of the skin to achieve optimal pharmacological effects.

**FIGURE 1:**
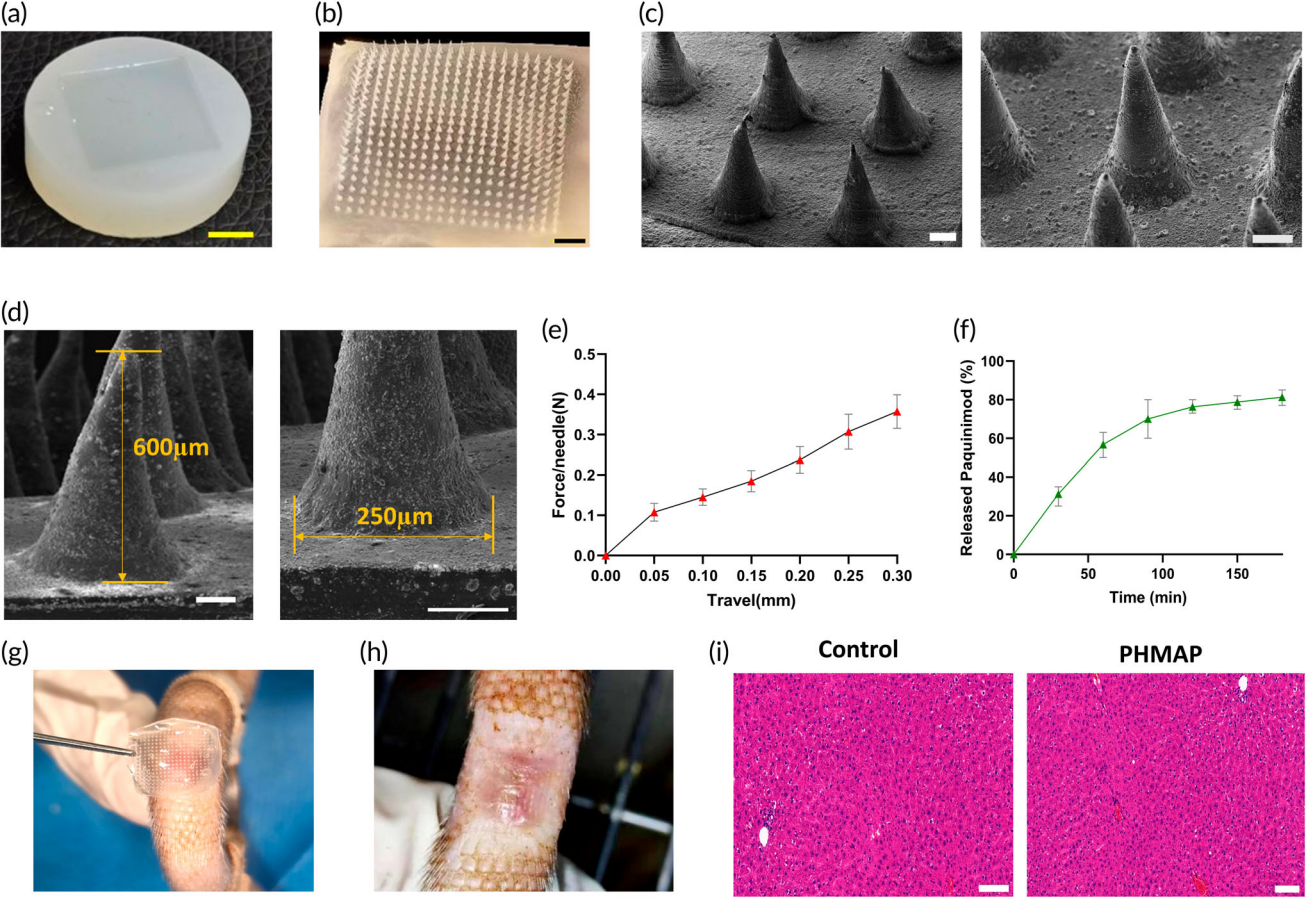
Synthesis and Characteristic of PHMAP. (a) Representative macroscopic image of the solution to mold. Yellow scale bar = 5 mm. (b) Representative macroscopic image of the completed PHMAP. Black scale bar = 2 mm. (c) Representative microscopic image of the PHMAP observed under electron microscope. White scale bar = 100 μm. (d) The size demonstration of PHMAP observed under electron microscope. White scale bar = 100 μm. (e) Representative force‐displacement curves of the PHMAP of the compressive test (*n* = 4). (f) Drug release profile of PHMAP in PBS. Concentration = 500 μg/ PHMAP (*n* = 4). (g) Representative photograph of transdermal administration of PHMAP. (h) Representative photographs of HS puncture after PHMAP administration for 3 days. (i) Representative HE stained sections of the rat liver after control and PHMAP administration. White scale bar = 100 μm.

Compared with traditional metal‐ and silicone‐based microneedles, HAMA‐based Hybrid microneedles have a larger drug‐carrying capacity and better biocompatibility,^47^ which can dissolve deep into the dermis without dissolving, and possess a more extended and more stable drug release.^48^ In this study, we prepared the HAMA‐based microneedles by dissolving HAMA in a 0.5 mg/mL Paquinimod PBS solution to create a hydrogel precursor. We added 0.6 mL of this solution to a mold (Figure 1a), followed by negative pressure defoaming at room temperature, and then heated and concentrated at 35°C for 6 h. A second 0.4 mL drop of the hydrogel precursor was added and subjected to a second 6‐h heating and concentration at 35°C. The mixture was then exposed to 405 nm ultraviolet light for 5 s to cure. Finally, 20% (w/v) PVA solution was applied as a composite base film, which was heated and dried at 35°C for 10 h or at room temperature overnight before demolding (Scheme 1). The PHMAP consists of a square array of 20 × 20 needles with a needle spacing of 550 μm and a coefficient of variation below 10%, indicating structural uniformity. Forty independent fabrication runs under identical conditions yielded highly reproducible results. Scanning electron microscopy (SEM) images of multiple batches confirmed consistent geometric features. (Figure 1b,c). To bypass the stratum corneum barrier, achieve dermal penetration, and enhance drug permeability for precise macrophage targeting, we designed the PHMAP with a needle height of 600 μm, a needle base diameter of 250 μm, and a patch size of approximately 1.5 × 1.5 cm(Figure 1d). The force acting on PHMAP increases gradually with the displacement and shows good elasticity in this compressive experiment (Figure 1e). The finalized PHMAP each contained 500 μg of Paquinimod. The drug release rate achieved 80% after approximately 3 h of incubation in PBS at room temperature (Figure 1f). It can be directly applied to the surface of skin scars during treatment, providing precise and long‐lasting drug delivery (Figure 1g). Additionally, the patches could be easily inserted into the scar tissue of the rats (Figure 1h) and liver sampling demonstrated that the PHMAP exhibited a favorable safety profile (Figure 1i).

### 3.2 Paquinimod and HAMA Hydrogel materials exhibited ideal biocompatibility with non‐cytotoxicity

**FIGURE 2:**
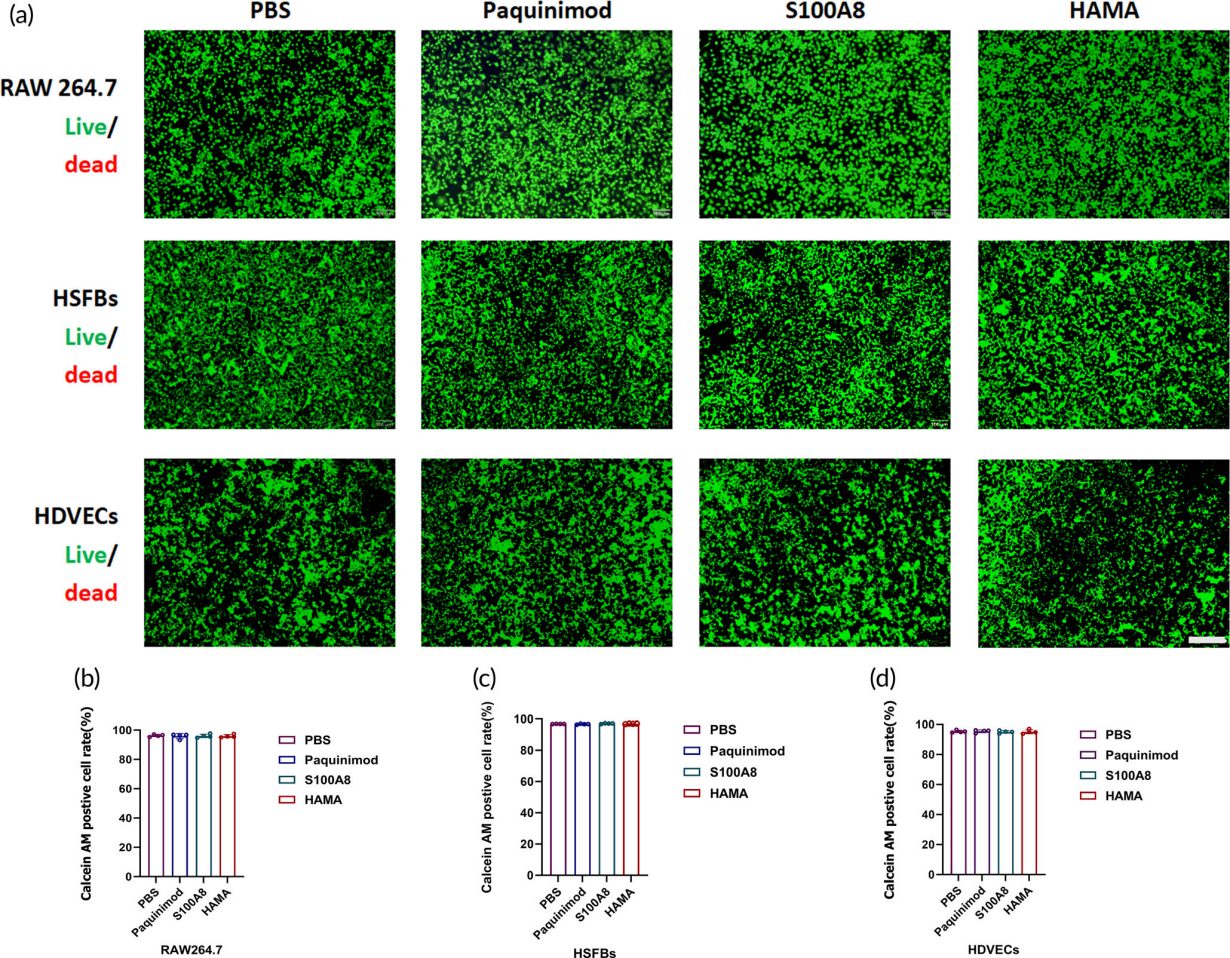
Assessment of the biocompatibility and cytotoxicity of Paquinimod and the HAMA hydrogel material. (a) Representative live/dead fluorescence images of Paquinimod (500 μg/mL) co‐cultured with macrophages (RAW264.7), fibroblasts (HSFBs), and vascular endothelial cells (HDVECs) on day 4, alongside controls (PBS, S100A8 [5 μg/mL], and HAMA [5% w/v]). white scale bar = 200 μm. (b–d) Quantification of live cell proportion on day 4 for Paquinimod (500 μg/mL) co‐cultured with RAW264.7, HSFBs, and HDVECs, as well as for controls (PBS, S100A8 [5 μg/mL], and HAMA [5% w/v]) (*n* = 4). Data are expressed as mean ± SD; One‐way analysis of variance (ANOVA). No significant difference.

To assess the cytotoxicity and biocompatibility of PHMAP, we co‐cultured Paquinimod and other raw materials with macrophages (RAW264.7), fibroblasts (HSFBs), and vascular endothelial cells (HDVECs) for 4 days. Live‐dead staining results indicated no significant difference in the proportion of live cells between the Paquinimod and control groups (Figure 2a–d), demonstrating that Paquinimod exhibits minimal toxicity and maintains good biocompatibility. To rule out confounding factors, HAMA hydrogel and S100A8 protein were also co‐cultured with RAW264.7, HSFBs, and HDVECs for 4 days, followed by live‐dead staining (Figure 2a–d). The results showed no significant differences compared to the Paquinimod and PBS groups, indicating that neither HAMA hydrogel nor S100A8 protein has any notable toxic effects on the cells.

### 3.3 Paquinimod did not directly inhibit the proliferation and migration of HSFBs and HDVECs

**FIGURE 3:**
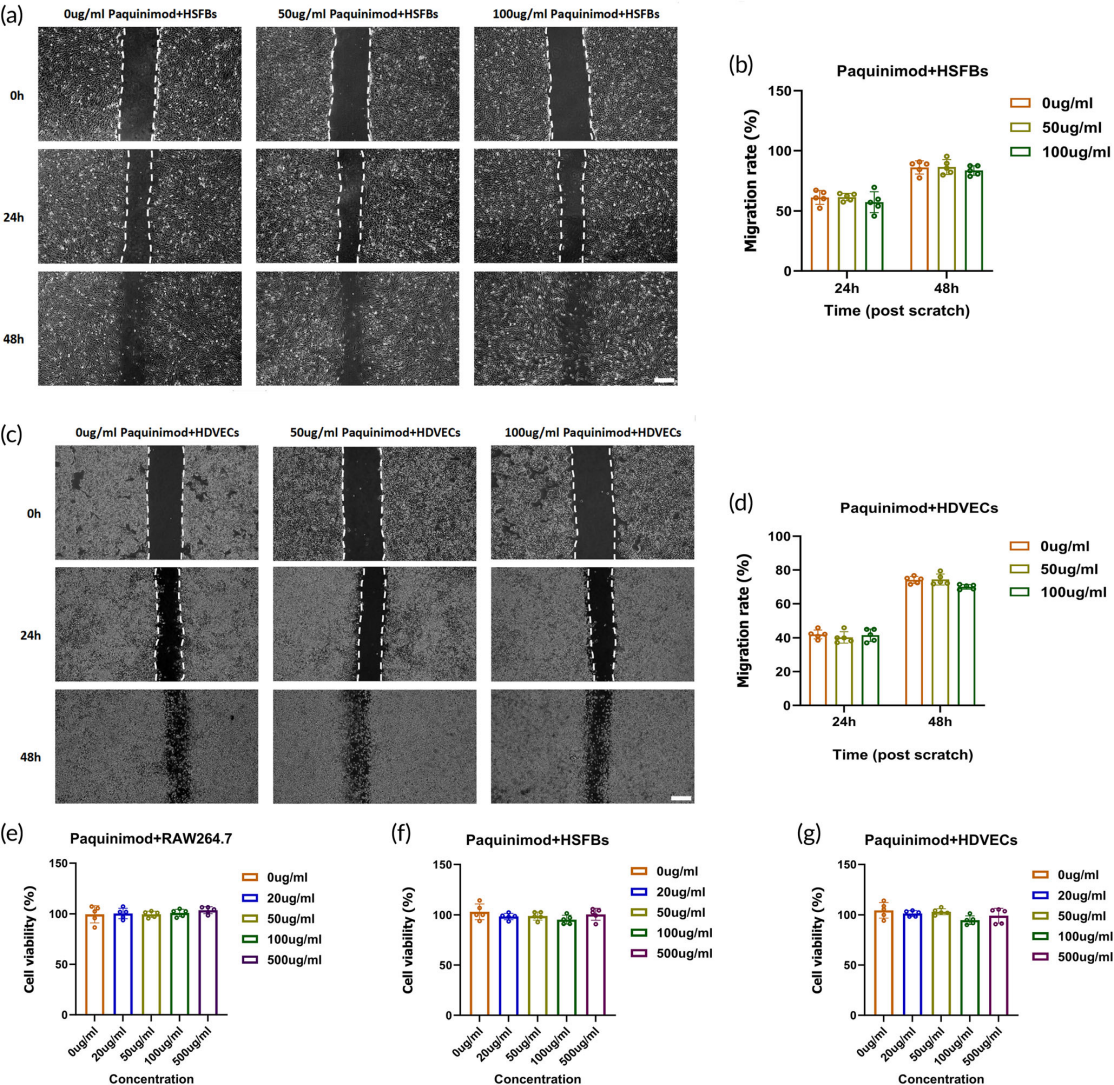
Paquinimod did not directly affect the migration of HSFBs and HDVECs. (a) Representative images of scratch assays showing fibroblast migration in the presence of Paquinimod at concentrations of 50 and 100 μg/mL. The control consists of an equal amount of PBS. White scale bar = 200 μm. (b) Quantification of HSFBs migration rate under different conditions (*n* = 5). (c) Representative images of scratch assays showing HDVECs migration with Paquinimod at concentrations of 50 and 100 μg/mL. Edges of the scratches are marked with a white line, and the control group contains only PBS. White scale bar = 200 μm. (d) Quantification of HDVECs migration rate in different groups (*n* = 5). (e–g) Quantitative results of CCK‐8 assays for cells co‐cultured with macrophages (RAW264.7), HSFBs, and HDVECs for 48 h at varying concentrations of Paquinimod (0, 20, 50, 100, and 500 μg/mL) (*n* = 5). Data are expressed as mean ± SD; One‐way analysis of variance (ANOVA). No significant difference.

To investigate the mechanism by which Paquinimod affects HSFBs and HDVECs, we co‐cultured these cells with various concentrations of Paquinimod. The migration rate of HSFBs and HDVECs was observed (Figure 3a–d). Although Paquinimod may have a mild inhibitory effect on HSFBs migration, the reduction in migration rate compared to the control group at 24 and 48 h was not statistically significant (Figure 3a,b). The effect of Paquinimod on HDVECs migration was similarly non‐significant (Figure 3c,d). CCK‐8 cytotoxicity assays further indicated that Paquinimod had no significant impact on cell growth (Figure 3e–g). Therefore, Paquinimod is not cytotoxic and has limited inhibitory effects on the proliferation and migration of both HSFBs and HDVECs.

### 3.4 Paquinimod inhibited macrophages polarization toward M1

**FIGURE 4:**
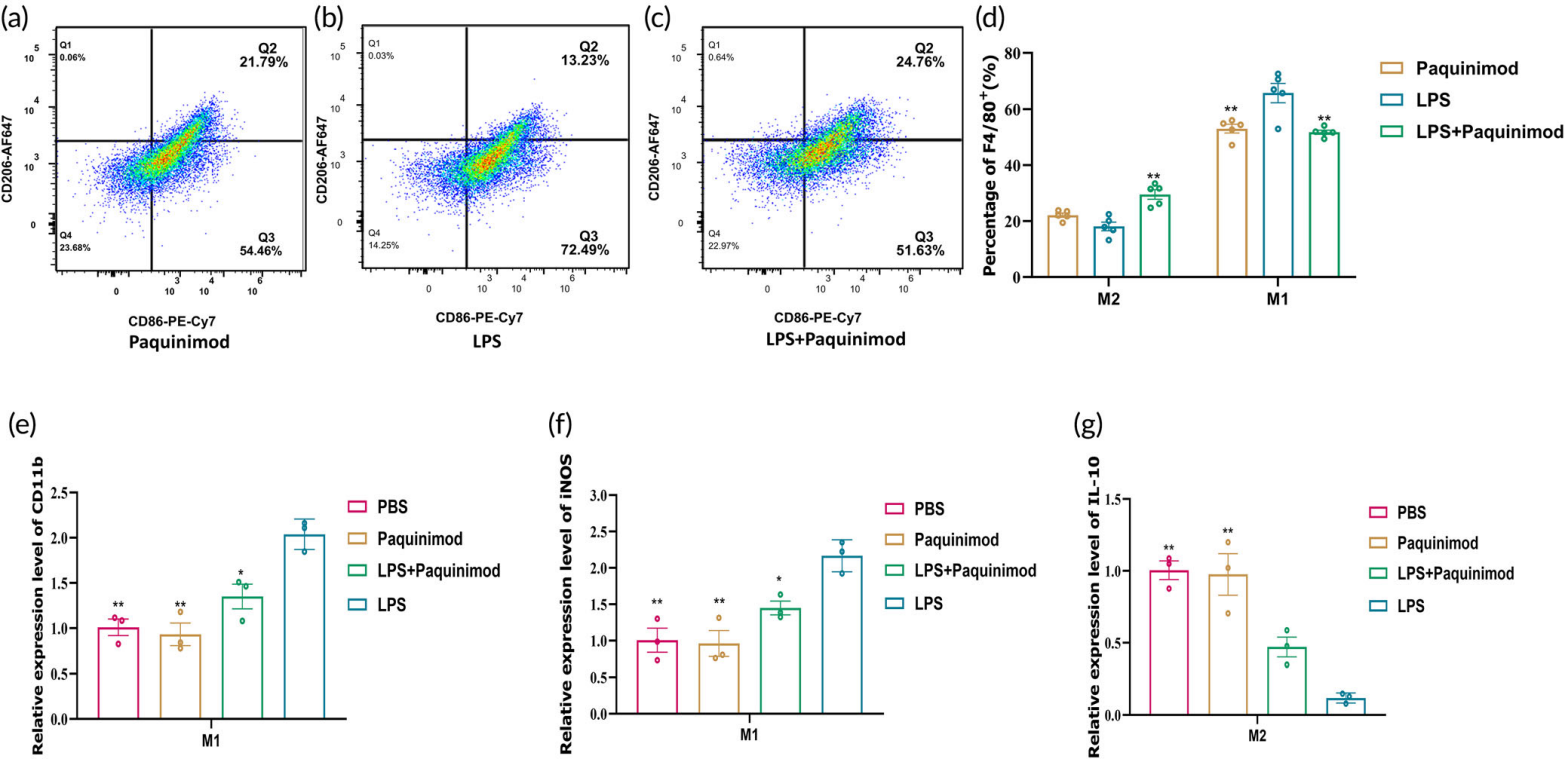
Paquinimod inhibited macrophages polarization toward M1. (a–c) Representative scatter plots from flow cytometry assays showing macrophage polarization after 24 h of co‐culture with Paquinimod (100 μg/mL) (a), LPS (100 ng/mL) (b), or both LPS (100 ng/mL) and Paquinimod (100 μg/mL) (c). (d) The F4/80 + CD86 + CD206− cell population was classified as M1‐type macrophages, and F4/80 + CD86 + CD206+, F4/80 + CD86 − CD206+ cell populations were categorized as M2‐type macrophages (*n* = 5). (e–g) Fluorescence quantitative qPCR for CD11b expression, (f) Fluorescence quantitative qPCR for iNOS expression, (g) Fluorescence quantitative qPCR for IL‐10 expression, the internal reference was GAPDH. Error bars represent the standard deviation from four independent experiments (*n* = 3). Data are expressed as mean ± SD, with statistical significance determined by two‐way and one‐way ANOVA (*p < 0.05, **p < 0.01).

To further investigate the immunomodulatory role of Paquinimod, we used LPS (100 ng/mL) to induce macrophage polarization toward M1.^49^ At the same time, the experimental group was added Paquinimod (100 μg/mL), while the other groups were treated with LPS (100 ng/mL) or Paquinimod (100 μg/mL) alone. After 24 h, flow cytometry was used to analyze the expression of surface markers such as CD86, CD206, and F4/80 (Figure 4a–c). The results showed that applying Paquinimod effectively inhibited macrophage polarization to M1(Figure 4d). The polarization results were further validated using a qPCR assay that employed CD11b and iNOS to identify M1 and IL‐10 to identify M2 (Table S1). These findings showed that Paquinimod significantly inhibited macrophage polarization to M1 (Figure 4e–g), indicating that it might influence HSFBs and HDVECs by modulating macrophage polarization, which in turn reduces collagen accumulation and neoangiogenesis.

### 3.5 Paquinimod‐treated macrophages inhibited the proliferation, migration, and collagen contraction of HSFBs

To test our hypothesis, we co‐cultured HSFB with macrophages (RAW264.7) treated with LPS (100 ng/mL), PBS, or both LPS (100 ng/mL) and Paquinimod (100 μg/mL) for 48 h. Scratch assays, collagen contraction assays, and CCK‐8 assays were used to detect cell migration, collagen contraction, and proliferation ability at 24 and 48 h.

**FIGURE 5:**
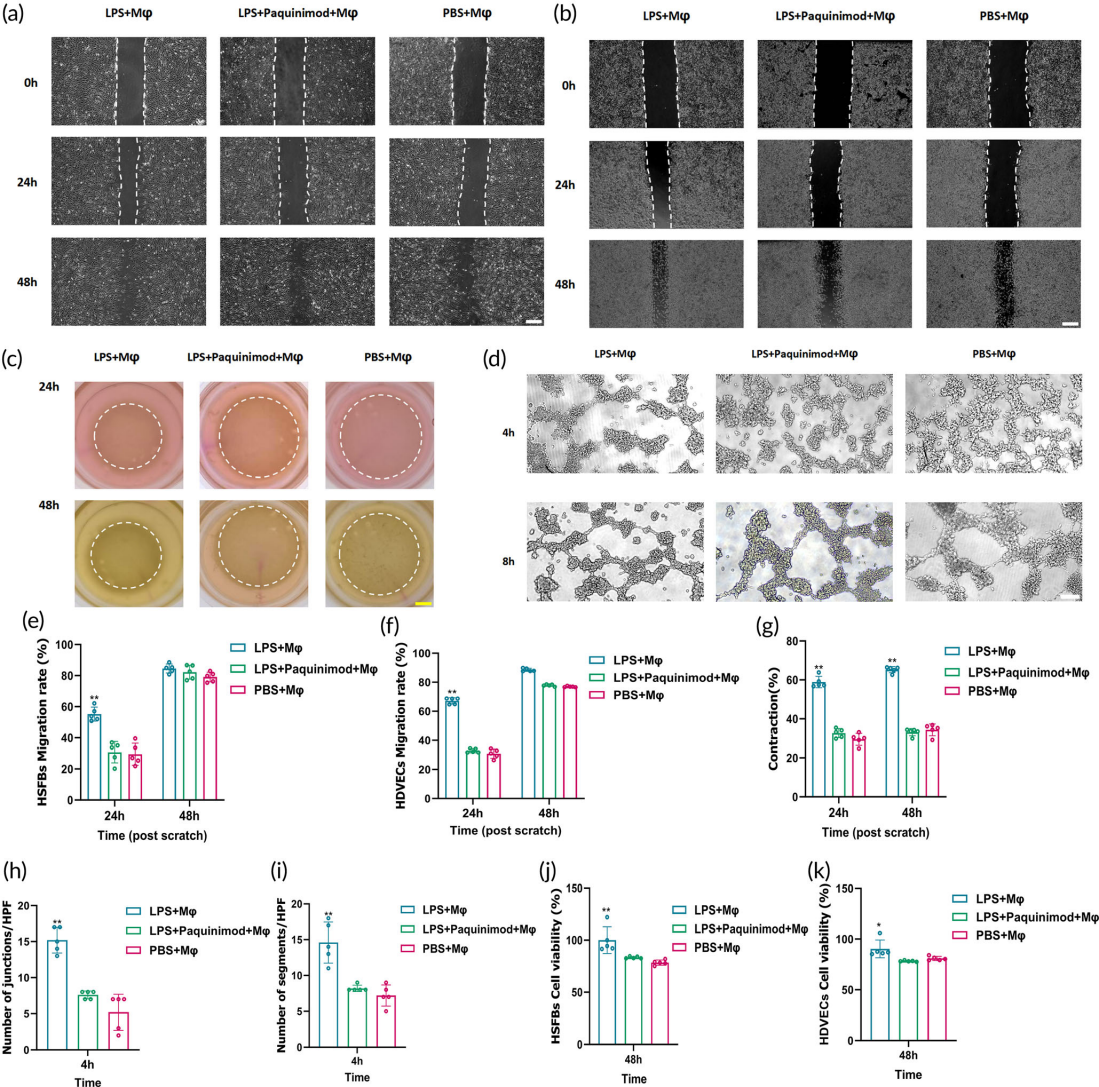
Paquinimod‐treated macrophages inhibited the proliferation, migration, and character of HSFBs and HDVECs. (a, b) Representative images of scratch assays showing the migration of HSFBs (a) and HDVECs (b) co‐cultured with macrophages (RAW264.7) treated with LPS (100 ng/mL), PBS or both LPS (100 ng/mL) and Paquinimod (100 μg/mL) for 48 h. The control group macrophages received PBS only. White scale bar = 200 μm. (c) Representative images of collagen contraction assays of HSFBs under different conditions for 48 h. Yellow scale bar = 2 mm. (d) Representative images of vascular tube‐formation assays showing the tube‐formation ability of HDVECs under different conditions for 4 and 8 h. White scale bar = 200 μm. (e, f) Quantification of HSFBs (e) and HDVECs (f) migration rate under different conditions (*n* = 5). (g–i) Quantification of collagen contraction (g) junctions (h) and segments (i) formed by HDVECs under different treatment conditions (*n* = 5). (j, k) Quantification of CCK‐8 assay for HSFBs (j) and HDVECs (k) co‐cultured for 48 h under different conditions (*n* = 5). Data are expressed as mean ± SD; two‐tailed unpaired Student's *t*‐test. Significantly differences are indicated by *p < 0.05 or **p < 0.01 versus the control (PBS + Mφ).

Results showed that HSFB co‐cultured with macrophages treated with LPS displayed a significant exhibition of migration (Figure 5a,e), collagen contraction (Figure 5c,g), and proliferation ability (Figure 5j) (*p* < 0.01). In contrast, macrophage products not stimulated by LPS and Paquinimod co‐cultured with HSFBs showed no significant difference in migration rates compared to the blank control group with PBS (Figure S1a).

### 3.6 Paquinimod‐treated macrophages inhibited the proliferation, migration, and angiogenesis of HDVECs

To investigate the effects of Paquinimod on HDVECs, we conducted experiments similar to those with fibroblasts. Macrophages were co‐cultured with Paquinimod, and the cell products were then co‐cultured with HDVECs for 48 h. We assessed cell migration rates at 24 and 48 h using scratch assays (Figure 5b,f) and cell proliferation at 48 h using CCK‐8 assays (Figure 5k). Angiogenesis assays demonstrated that Paquinimod and macrophage co‐culture significantly inhibited vascular tube formation (Figures 5d,h,i and S1c). Like the HSFBs, macrophage products not stimulated by LPS and Paquinimod co‐cultured with HDVECs showed no significant difference in migration rates compared to the blank control group with PBS (Figure S1b).

Since HSFBs and HDVECs play key roles in HS, effectively inhibiting their proliferation, migration, and biological functions is crucial.^50^ Based on these results, we conclude that Paquinimod can indirectly inhibit the proliferation and migration of both HSFBs and HDVECs by modulating macrophage activity.

### 3.7 Paquinimod alleviated the HS of rat tail

**FIGURE 6:**
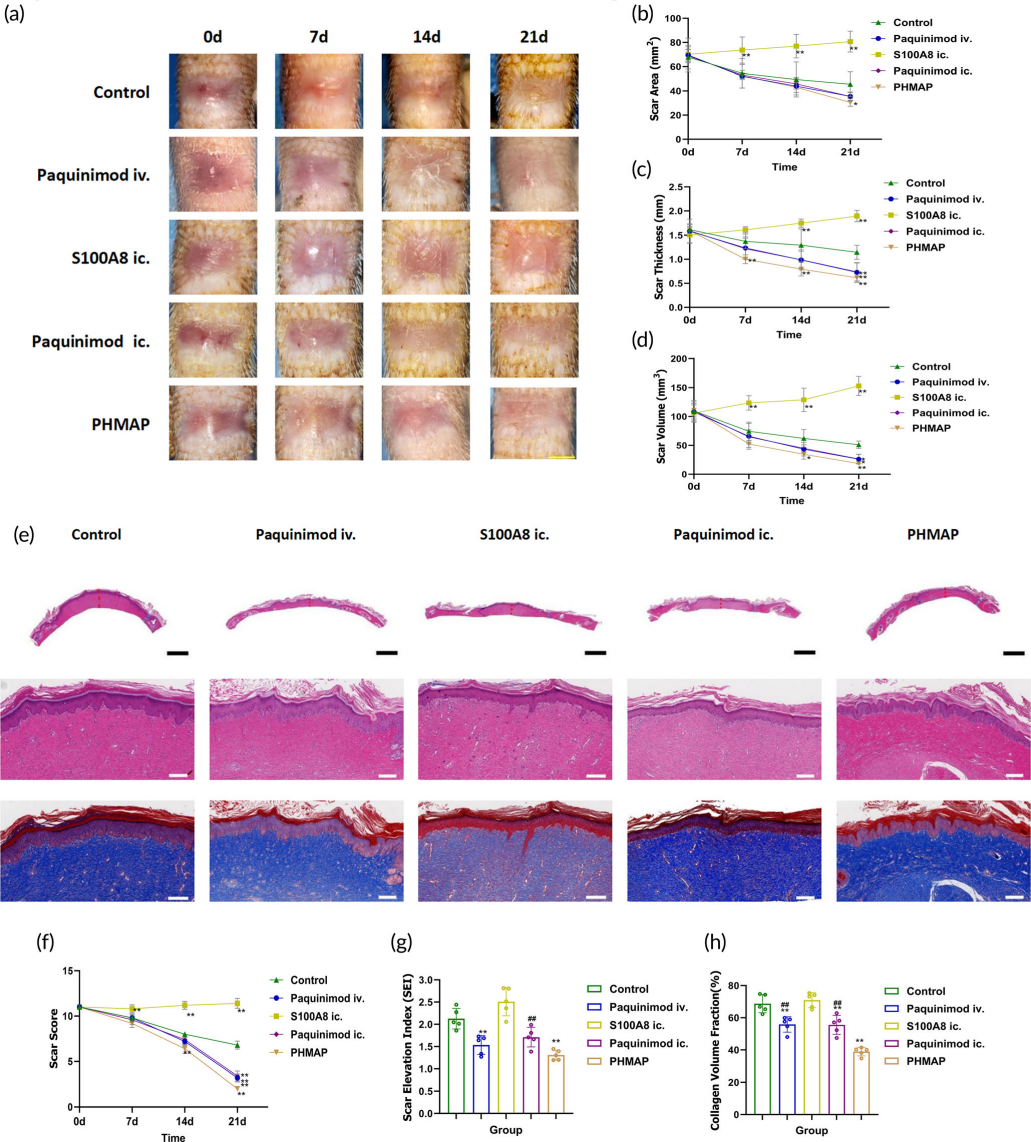
The PHMAP exhibited superior efficacy in treating HS. (a) Representative macroscopic images of rat tail HS in the control group, Paquinimod iv. group (Paquinimod vein injection), S100A8 ic. group (S100A8 intradermal injection), Paquinimod ic. group (Paquinimod intradermal injection), and PHMAP group (Paquinimod‐Hydrogel Hybrid Microneedle Array Patch) at days 0, 7, 14, and 21 after treatment. Yellow scale bar = 5 mm. (b–d) Quantification of Scar area (b), scar thickness (c), and scar volume (d) at 0, 7, 14, and 21 days post‐treatment in different treatment groups. (e) Representative HE and Masson staining images of rat tail HS tissue at day 21 post‐treatment. Red dashed line indicates scar thickness; blue dashed line indicates normal skin thickness around the scar. Black scale bar = 1 mm, white scale bar = 200 μm. (f) Quantification of scar fraction (VSS) at 0, 7, 14, and 21 days post‐treatment in different treatment groups (*n* = 7). (g, h) Quantification of Scar elevation index (SEI) (g) at day 21 post‐treatment, and collagen volume fraction (CVF) (H) (*n* = 5). Data are expressed as mean ± SD; two‐tailed unpaired Student's *t*‐test. Significantly differences are indicated by *p < 0.05 or **p < 0.01 versus the control group, and ^#^ *p* < 0.05 or ^##^ *p* < 0.01 versus the PHMAP group.

In HS and keloid studies, S100A8/A9 expression was significantly upregulated during skin injury and wound healing, and immunohistochemistry of both human and rat normal skin with HS pathology showed the same results (Figure S2a,b). Its downstream receptor TLR4 was also significantly increased in HS (Figure S3a,b). In order to verify the therapeutic effects of Paquinimod in vivo, we used the rat tail HS model in SD rats.^51^ The rat tail was created using a scalpel to create about a 0.8*0.8 cm trauma, with the metal ring fixation of the rat tail to maintain constant tension.^52^ After 3 weeks, when the HS formed, rats in the experimental group were injected via the tail vein with 1 mL of a 0.5 mg/mL Paquinimod solution, while the control group received 1 mL of PBS. Treatment was repeated at 7 and 14 days. Photographs were taken at 0, 7, 14, and 21 days (Figure 6a), and samples were taken at 21 days. Immunohistochemical staining results show Paquinimod certainly selectively targets S100A8/A9 (Figure S4a,b). Compared to the control group, there was no significant difference in the reduction of scar area in the experimental group (Figure 6b). However, the thickness of the scar was significantly reduced (Figure 6c), and its color visibly faded. There was a notable decrease in blood supply, with the scar becoming flatter and softer and deformable under pressure (Figure 6d,e). Additionally, the scar scores (VSS) showed significant improvement compared to the control group (Figure 6f). Quantification of Scar elevation index (SEI) and collagen volume fraction (CVF) also shows the inhibitory effects of Paquinimod(Figure 6g,h).

In summary, Paquinimod can effectively inhibit the inflammatory cascade response and improve themicroenvironment.

### 3.8 S100A8 enhanced scar proliferation

Additionally, to verify the promoting effect of S100A8 on HS and to support the mechanism by which Paquinimod inhibits HS, we injected 0.1 mL of a 50 μg/mL S100A8 PBS solution into the scar. This treatment significantly (*p* < 0.01) promoted HS progression (Figure 6b–d) and sustained proliferation throughout the 21‐day observation period (Figure 6f). This result not only verified the hypothesis that S100A8 could promote scar proliferation but also solved the problem of the slow decrease in scar thickness and volume of murine caudal scar over time, which provided a new direction for the construction of animal models of HS.

### 3.9 PHMAP exhibited superior efficacy in HS treatment

In order to further compare the therapeutic effects of different modes of drug administration, we still used the rat tail HS model in SD rats and injected 0.1 mL (5 mg/mL) of Paquinimod PBS solution into the scar at 0 days, while the experimental group used the transdermal administration of PHMAP containing 500 μg of the drug, and the administration of the drug was repeated twice at 7 and 14 days to compare the therapeutic effects of the PHMAP, intra‐scar injection, tail vein injection, and other modes of drug administration (Figure 6a). Gross visual observation revealed that the PHMAP treatment group had significant advantages in reducing scar thickness (Figure 6c), scar volume (Figure 6d), and scar score (VSS) (Figure 6f) (*p* < 0.01). To further validate the therapeutic effect at the histologic level, we performed HE and Masson staining on HS specimen sections at 21 days (Figure 6e). Consistent with the macroscopic observations, histological analysis showed that the PHMAP treatment group had the lowest scar proliferation index (SEI) (Figure 6g) and collagen volume fraction (Figure 6h) (*p* < 0.01). The efficacy was significantly superior to that of both the intra‐scar injection and tail vein injection groups, suggesting that the PHMAP has promising potential for clinical application in HS treatment.

### 3.10 PHMAP reduced M1 macrophage polarization and inhibited Neovascularization in HS

**FIGURE 7:**
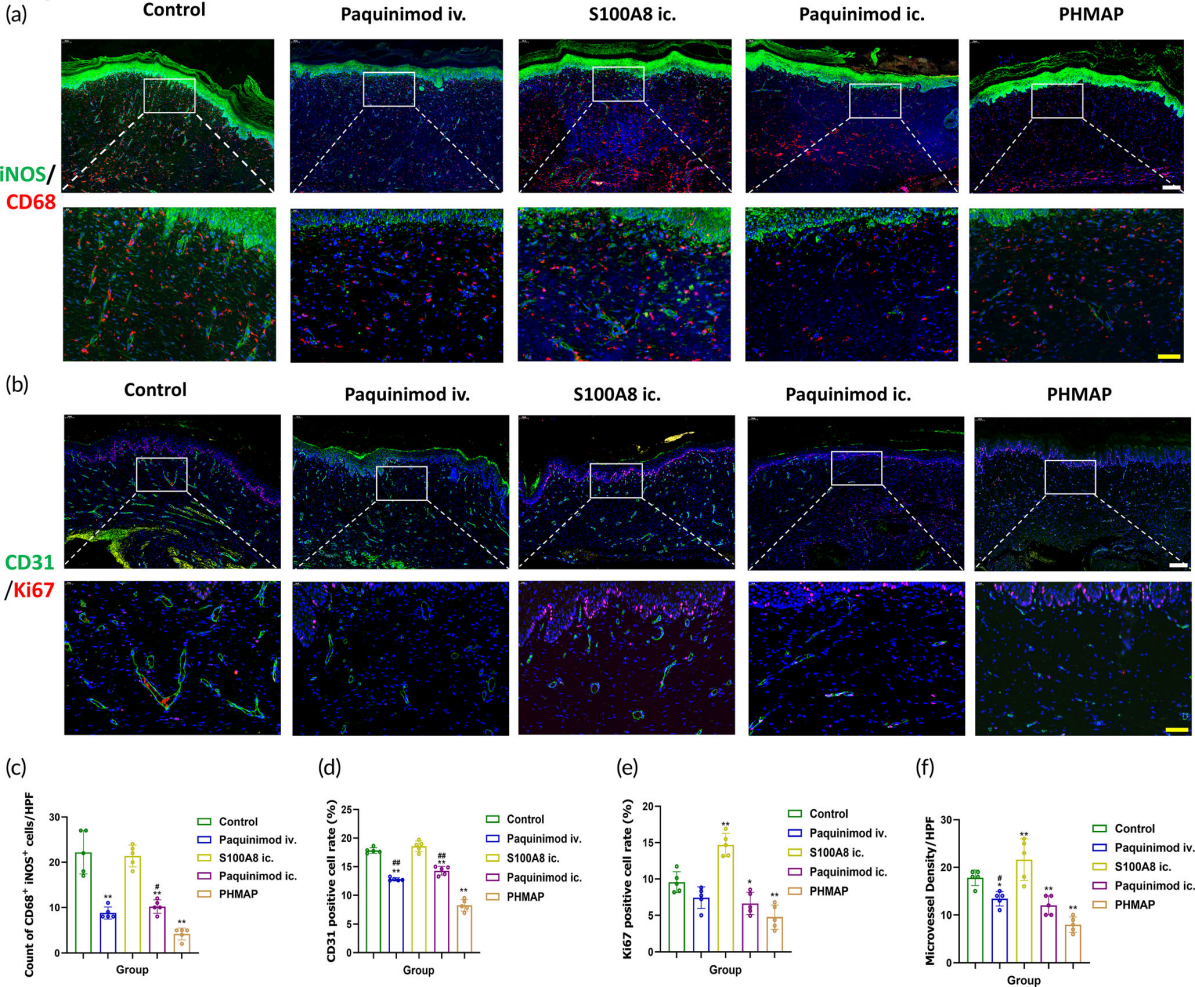
PHMAP reduced M1 macrophages polarization and inhibited Neovascularization in HS. (a) Representative images of M1 (CD68 + iNOS+) double immunofluorescence staining on day 21, showing CD68 (red) and iNOS (green). White scale bar = 200 μm, yellow scale bar = 50 μm. (b) Representative images of VECs double immunofluorescence staining images on day 21 showing Ki67 (red) and CD31 (green). White scale bar = 200 μm, yellow scale bar = 50 μm. (c) Quantification analysis of M1 (iNOS/CD68 double‐positive cells) in different treatment groups (*n* = 5). (d, e) Quantification analysis of the CD31‐positive cells (d), Ki67‐positive cells (e) proportion in different treatment groups (*n* = 5). (f) Quantification analysis of the Microvessel density (MVD) in different groups (*n* = 5). Data are expressed as mean ± SD; two‐tailed unpaired Student's *t* test. Significantly differences are indicated by *p < 0.05 or **p < 0.01 versus the control group, and ^#^ *p* < 0.05 or ^##^ *p* < 0.01 versus the PHMAP group.

In order to validate the mechanism of action of PHMAP at the histologic level, we observed a significant reduction in double‐stained M1‐type macrophages, identified using CD68 and iNOS labels, in 21‐day scar tissue sections from the Paquinimod‐treated group (Figure 7a,c). To quantitatively assess Paquinimod's inhibitory effects on cell proliferation and neovascularization, we used fluorescent labeling for CD31 and Ki67(Figure 7b). CD31 labeling revealed that PHMAP more effectively inhibited neovascularization compared to other administration methods (*p* < 0.01) (Figure 7d), the MVD quantitative results also prove this(Figure 7f), with no significant difference between intrascar and caudal vein injection methods. Ki67 staining also confirmed that Paquinimod significantly reduced cell proliferation within the scar (Figure 7e).

### 3.11 PHMAP reduced collagen deposition in HS

**FIGURE 8:**
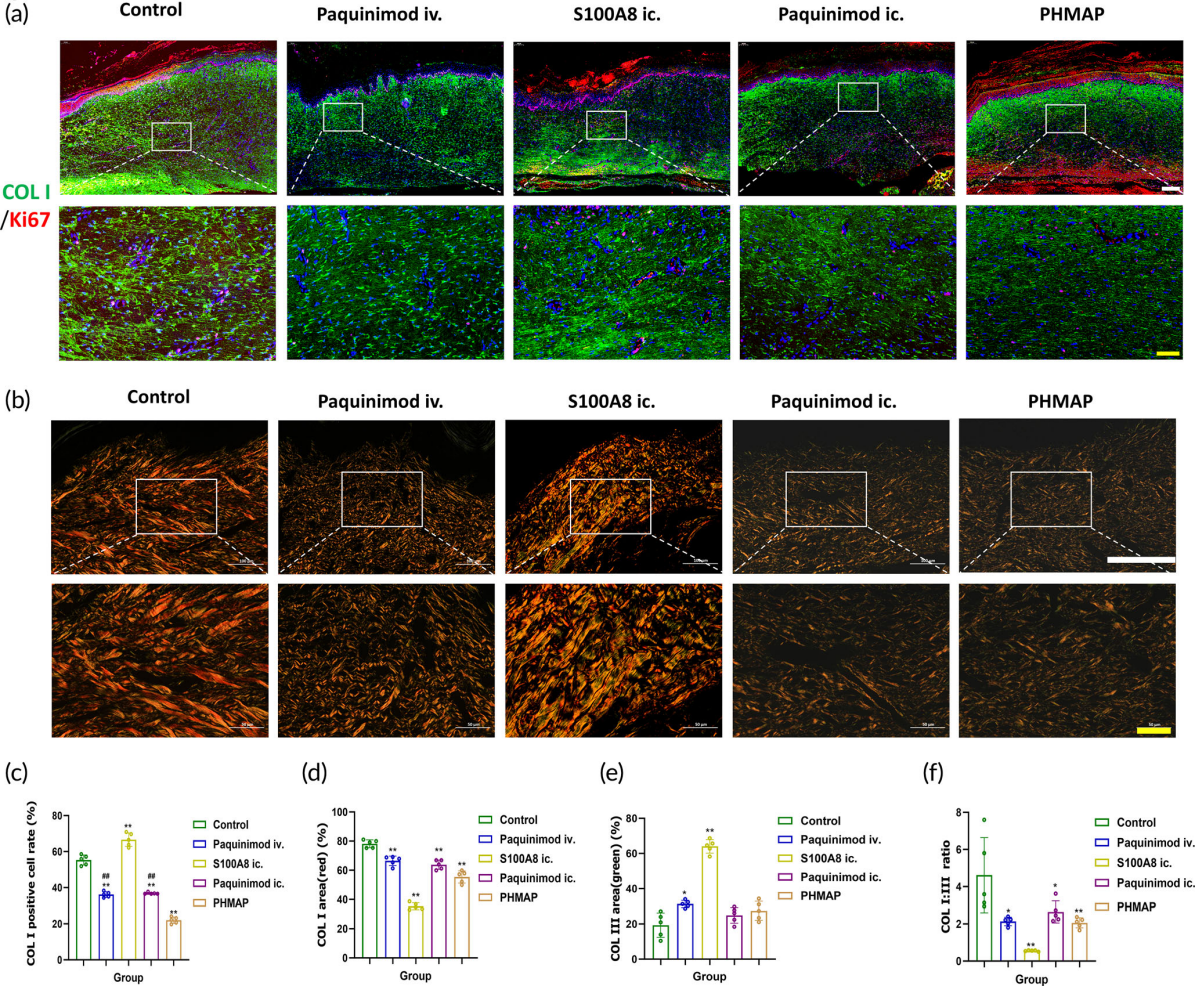
PHMAP reduced collagen deposition in HS. (a) Representative immunofluorescence staining images of different treatment groups on day 21, ki67 (red)/Collagen I (green), white scale bar = 200 μm, yellow scale bar = 50 μm. (b) Representative images of Sirius red staining of different treatment groups. White scale bar = 200 μm, yellow scale bar = 50 μm. (c) Quantification analysis of the Collagen I‐positive cells proportion in different treatment groups (*n* = 5). (d, e) Quantification analysis of the area proportion of collagen I (d) and collagen III (E) (*n* = 4). (f) The ratio of collagen I/III in different groups (*n* = 5). Data are expressed as mean ± SD; two‐tailed unpaired Student's *t* test. Significantly differences are indicated by *p < 0.05 or **p < 0.01 versus the control group, and ^#^ *p* < 0.05 or ^##^ *p* < 0.01 versus the PHMAP group.

We similarly validated the significant role of massive collagen accumulation in HS, emphasizing its importance for assessing therapeutic efficacy. Immunofluorescence staining of type I collagen fibers and Sirius red staining further demonstrated that Paquinimod significantly inhibited collagen accumulation in HS (Figure 8a,b). PHMAP treatment was more effective than intravenous and intradermal injections in reducing collagen accumulation (Figure 8c). Quantitative analysis of Sirius red staining revealed a significant reduction in the percentage of type I collagen area in the treatment group receiving Paquinimod. Conversely, the percentage of type III collagen area increased, resulting in a decreased ratio of type I to type III collagen (Figure 8d–f). However, in the S100A8 ic. group, there was a significant accumulation of type III collagen, which we propose is linked to its role in enhancing scar proliferation. This accumulation suggests that the scar in the S100A8 group resembles the microstructure of the newly created scar more closely.

## 4 CONCLUSION

In conclusion, this study validated that Paquinimod is a safe, non‐toxic, and biocompatible therapeutic agent for HS. It suppresses the proliferation, migration, collagen synthesis, and angiogenesis of HSFBs and HDVECs by inhibiting M1 polarization in the HS microenvironment. Based on this critical mechanism, we further developed the PHMAP, which exhibited superior efficacy to traditional intravenous and intralesional injections. This precise, long‐lasting, novel treatment approach for HS demonstrates promising clinical application potential.

## AUTHOR CONTRIBUTIONS

**Zihui Zhang:** Conceptualization; methodology; writing – original draft; writing – review and editing; formal analysis; validation; data curation. **Peng Wang:** Conceptualization; methodology; writing – original draft; investigation; validation; resources; project administration; funding acquisition. **Hengdeng Liu:** Investigation; validation. **Hanwen Wang:** Validation; formal analysis. **Miao Zhen:** Methodology; validation. **Xuefeng He:** Investigation; formal analysis. **Suyue Gao:** Visualization; software. **Juntao Xie:** Project administration; supervision; resources. **Julin Xie:** Resources; project administration; supervision; writing – review and editing; conceptualization; funding acquisition.

## CONFLICT OF INTEREST STATEMENT

The authors declare no conflict of interests.

## Supporting information


**DATA S1.** Supporting Information.

## Data Availability

The data that support the findings of this study are available from the corresponding author upon reasonable request.
