# Supplementary material for: Paquinimod‐hydrogel hybrid microneedle array patch alleviates hypertrophic scar via inhibiting M1 polarization
Source: Bioeng Transl Med. 2025 Mar 15;10(5):e70016. doi: 10.1002/btm2.70016 (PMC12478444; doi:10.1002/btm2.70016)
Supplement: Supplementary file 1 — DATA S1. Supporting Information. [file BTM2-10-e70016-s001.docx]

Supporting information

**Paquinimod‐hydrogel hybrid microneedle array patch alleviates hypertrophic scar via inhibiting M1 polarization**

Zihui Zhang, Peng Wang, Hengdeng Liu, Hanwen Wang, Miao Zhen, Xufeng He, Suyue Gao, Juntao Xie, Julin Xie*

Z. Zhang, P. Wang, H. Liu, H. Wang, M. Zhen, X. He, S. Gao, J. Xie

Department of Burn and Wound Repair Surgery, The First Affiliated Hospital of Sun Yat-sen University

No.58, Zhongshan 2nd Road, Guangzhou, 510080, China

Email: [xiejulin@mail.sysu.edu.cn](mailto:xiejulin@mail.sysu.edu.cn)


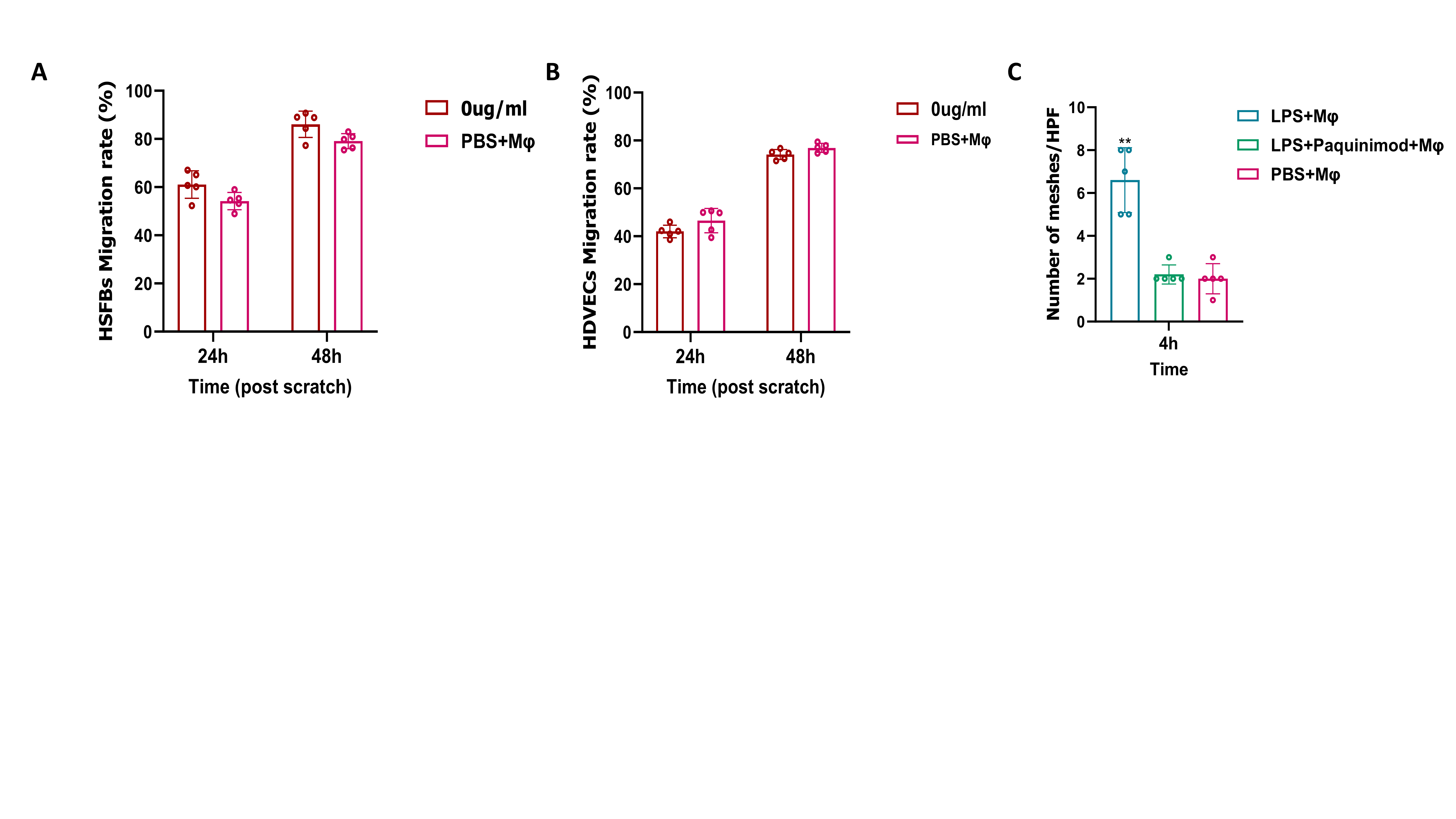


**Figure S1** Without LPS stimulation, Paquinimod-treated macrophages did not affect HSFBs and HDVEC migration compared to the PBS control. A)HSFBs and B) HDVECs migration was quantified in the 0ug/ml group (PBS) and the PBS+Mφ group (PBS with macrophage co-culture product) (n=5). C)Quantification of meshes formed by HDVECs under different treatment conditions (n = 5)

**
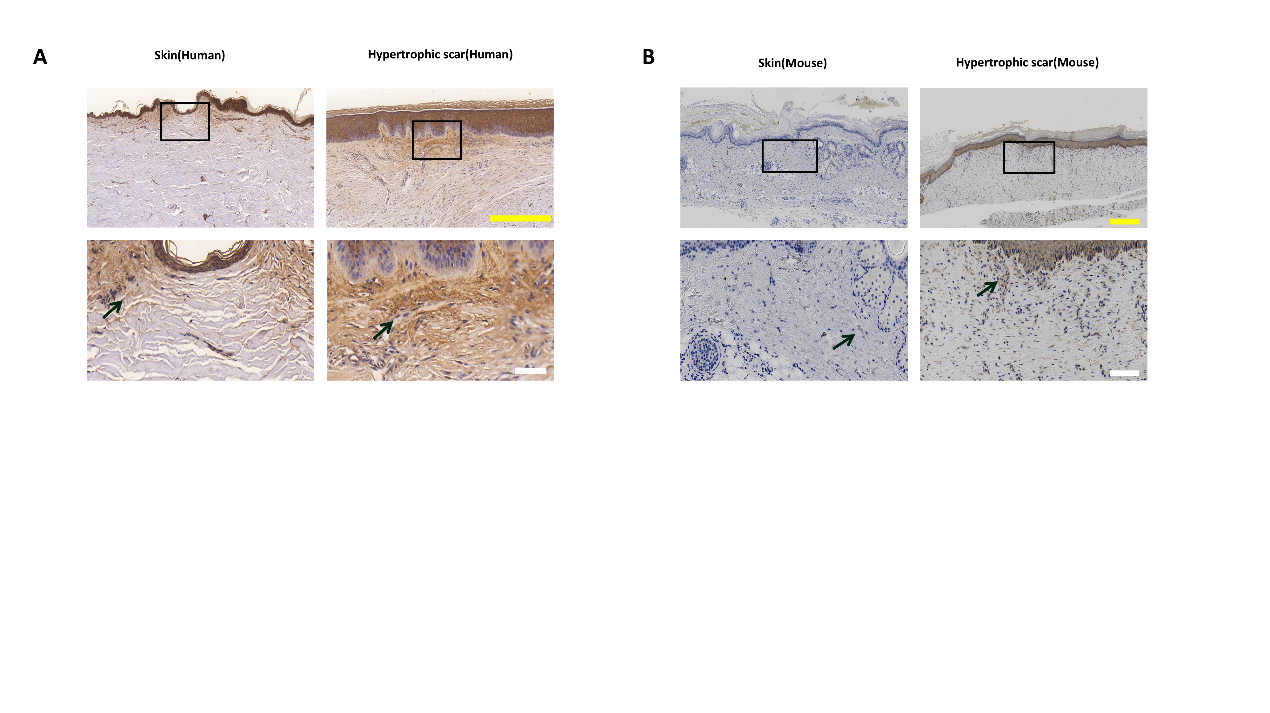
Figure S2** S100A8/A9 expression upregulates in HS. A) Human normal skin and HS S100A8(brown) immunohistochemical staining. Yellow scale bar =500µm. White scale=100µm. B) Rat tail normal skin and HS S100A8 (brown) immunohistochemical staining. Yellow scale bar = 500µm. White scale bar = 100µm.


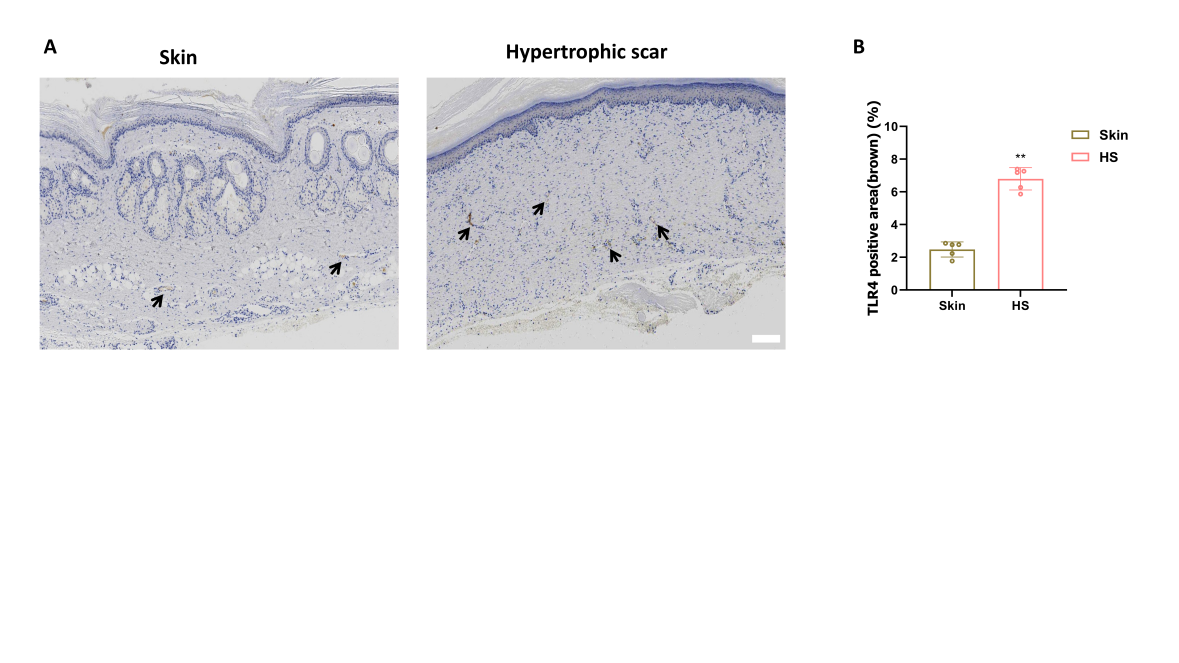


**Figure S3** TLR4 was significantly increased in HS. A) Rat normal skin with HS labeled by TLR4(brown) immunohistochemical staining. White scale bar= 200 µm. B) Proportion of TLR4-positive cells(brown) in different treatment groups(n=5). Statistical significance was determined by t-test, * p < 0.05 or ** p < 0.01 indicates a significant difference compared with the skin group.


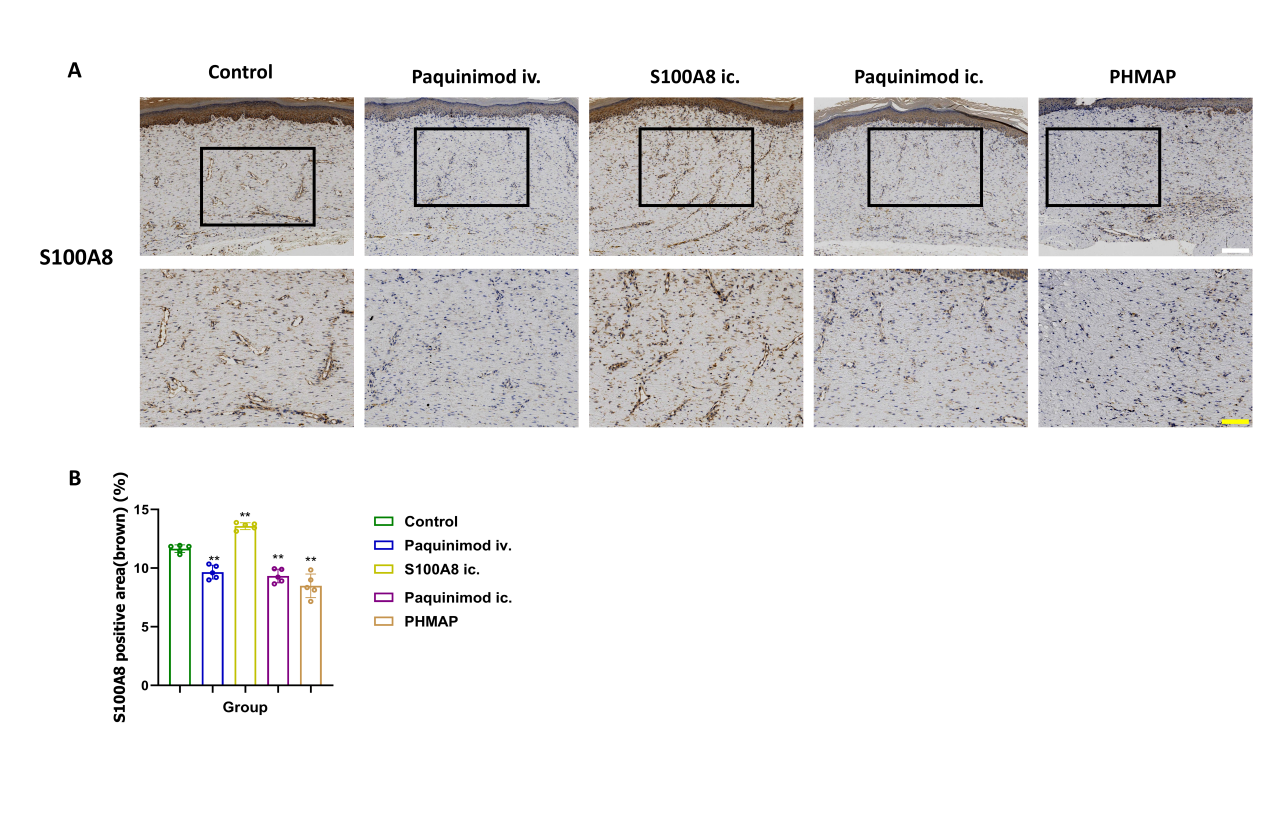


**Figure S4** Paquinimod selectively target S100A8/A9. A) Representative S100A8(brown) immunohistochemical staining images of different treatment groups on day 21, white scale bar = 200µm, yellow scale bar= 100µm. B) Proportion of S100A8-positive cells(brown) in different treatment groups(n=5). Statistical significance was determined by one-way ANOVA, * p < 0.05 or ** p < 0.01 indicates a significant difference compared with the control group.

**Table S1.** qRT-PCR primer information and sequences

| Primer information | Sequences (5'-3') | Length (bp) | Temperature (℃) |
| --- | --- | --- | --- |
| M-GAPDH-S | CCTCGTCCCGTAGACAAAATG | 133 | 60 |
| M-GAPDH-A | TGAGGTCAATGAAGGGGTCGT |  | 60 |
| M-CD68-S | GCCCAAGGAACAGAGGAAGACT | 194 | 60 |
| M-CD68-A | GTGGTGGCAGGGTTATGAGTG |  | 60 |
| M-CD11B-S | GTGAATATGTCCTTGGGCCTGTC | 136 | 60 |
| M-CD11B-A | GGTTGGAGCCGAACAAATAGC |  | 60 |
| M-Adgre1-S | GAGCAGATACAGCAATGCCAAG | 228 | 60 |
| M-Adgre1-A | CAGCAAGATGGTGCCCAGAGT |  | 60 |
| M-IL10-S | AATAAGCTCCAAGACCAAGGTGT | 81 | 60 |
| M-IL10-A | CATCATGTATGCTTCTATGCAGTTG |  | 60 |
| M-INOS-S | CTGTCGCAGCTCCCTATCTT | 166 | 60 |
| M-INOS-A | TCAGGTTCCTGATCCAAGTGC |  | 60 |
| M-MRC1-S | CAGGAGGACTGCGTGGTTATG | 211 | 60 |
| M-MRC1-A | GGTTTGCATCAGTGAAGGTGG |  | 60 |
| M-Cd86-S | TGGGCTTGGCAATCCTTATCTT | 141 | 60 |
| M-Cd86-A | CCAGCTCACTCAGGCTTATGTTT |  | 60 |
| M-Cd163-S | AGGAAACCAATCCCAGACACTA | 135 | 60 |
| M-Cd163-A | CGACCACCTCCACCTACCAA |  | 60 |
